# Current News and Opinion

**Published:** 1882-12

**Authors:** 


### CURRENT NEWS AND OPINION.

Dr. HENRY DRAPER died in this city on the 20th of November, being only forty-six. He was a son of the late Dr. Jno. W. Draper. In this case the mantle becomingly fell upon the son's shoulders.

Dr. Henry Draper, as a chemist and astronomer, will be missed by the scientific world. In honor of his scientific usefulness the Congress of the United States presented him with a gold medal, upon which was appropriately inscribed: "He adds lustre to ancestral glory."

The Bogus Bellevue College of Massachusetts falls properly into the hands of law. C. J. Eastman, Dean, and Noyes, president, have been arrested. Many diplomas have been sold—home and abroad—under a certificate of incorporation from the State bearing date May, 1880.

The Faculty of Medicine, in Paris, propose to create a higher degree than that of M. D. What degree will our Post-Graduate and Polyclynic Schools of New York confer?

Dr. J. Solis Cohen has been elected Honorary Prof. of Laryngology in Jefferson Medical College.

Dr. E. Evetzky, formerly of this city, and known as a talented and successful physician, removed to Durango, Col., some months ago. There he entered into a professional copartnership with a Dr. H. A. Clay. On the 3d instant he entered their office and shot Dr. Clay fatally, and instantly blew out his own brains. No difficulty was known to exist between them until the day previous, when Dr. Evetzky received a severe cowhiding from two females, who accused him of talking about them. He suspected Dr. Clay of having caused the trouble, which doubtless caused the tragedy.

A Stupid Town.—Certain citizens of Red Bank, N. J., have begun an action for conspiracy and libel against John H. Cook, editor of the Red Bank *Register*, for publishing certain statements regarding the prevalence of typhoid fever. The principal matter in dispute seems to be the number of cases said to be typhoid fever, which it is alleged, were overstated by Mr. Cook, it being held by the citizens of the town that there was only one case, and that an imported one, and that the other cases were "typho-malaria." When the strength of the case depends on a fever being "typho-malarial" instead of typhoid, it would be far wiser to clean out the privies than go to law.—*Med. Record.*

Suffocation by Illuminating Gas.—From July 1st, there have been fourteen deaths from the escape of illuminating gas in bed-rooms. Most of them have resulted from blowing out the gas.

Ovariotomy in an Infant.—Dr. Hingston, of Montreal, recently removed an ovarian tumor from a child two years of age. The patient was doing well at last account.—*Ex.*

A Practitioner in Texas (fortunately irregular) is said to have diagnosed a case of small-pox as "erysipelas from the toes to the knees, measles from the knees to the waist, and seven years' itch from the waist to the top of the head."—*Exehange*

An Anti-Tobacco Law.—A bill has been presented to the Michigan Legislature forbidding the use of tobacco by boys. The *Free Press* now urges the passage of an "Anti-pie-Law," to prevent the "enormous and growing evil" of eating too much pie.—*Exchange.*

A Wholesale Chemist in London manufactures one ton of homœopathic globules annually.

